# 

**Published:** 1891-10

**Authors:** 


### CONTENTS—OCTOBER, 1891.—VOL. VII, No. 4.

ORIGINAL COMMUNICATIONS—

The Aurora Epidemic. Letter from Dr. E. J. Beall, in reply to Criticism ...... 116

A Case of Circumscribed Dropsy. D. B. McGee, M. D. 137

SOCIETY NOTES—

Medical Alliance Transactions. Wood County Medical Society. S. S. and G. Association. N. W. Texas Medical Society, etc ...... 137-8

EDITORIAL—

Exploits in Medicine ...... 139

A Secretary of Public Health ...... 142

Regulating the Practice ...... 144

As Clear As Mud ...... 146

A New Feature in Medical Teaching ...... 147

MEDICAL NEWS AND MISCELLANY ...... 148

ADVERTISERS' NOTES, ETC ...... 153

BOOK NOTICES ...... 156

